# Endoscopic Synergy: Endoscopic Combined Intrarenal Surgery (ECIRS)-Guided “Cut-to-Light” Holmium Laser Retrograde Endoureterotomy in Ureteral Stricture Management

**DOI:** 10.7759/cureus.79758

**Published:** 2025-02-27

**Authors:** Suryaram Aravind, Punith Jain R, Velmurugan Palaniyandi, Hariharasudhan Sekar, Sriram Krishnamoorthy

**Affiliations:** 1 Urology, Sri Ramachandra Institute of Higher Education and Research, Chennai, IND; 2 Urology and Renal Transplantation, Sri Ramachandra Institute of Higher Education and Research, Chennai, IND

**Keywords:** endo urology, holmium laser, ho:yag, ureteral stricture, ureteral stricture management

## Abstract

This is a case report of a 50-year-old diabetic woman with chronic obstructive pulmonary disease who had an incidentally detected right-sided ureteral stricture. Ureteral strictures are a serious condition that may sometimes progress silently, ultimately resulting in ipsilateral renal function loss. Proper timing and appropriate treatment are essential to preserve renal function and prevent further complications. The management of long-segment strictures ranges from open repair to laparoscopic, robotic, and interventional techniques. This manuscript presents a case of a long-segment ureteral stricture successfully treated using a minimally invasive endourological technique, preserving normal urinary flow. This is the first study to report a successful synergistic interventional approach for the management of complex ureteral strictures.

## Introduction

Ureteral strictures are a serious condition characterized by a narrowing of the ureteral lumen that leads to functional kidney obstruction. This condition may sometimes progress silently, resulting in ipsilateral renal function loss. Proper timing and appropriate treatment are essential to preserve renal function and prevent further complications [1].

The management of long-segment strictures is a topic of considerable debate, with options ranging from open repair to laparoscopic, robotic (renal descensus, pyeloplasty, transureteroureterostomy (TUU), buccal mucosal graft ureteroplasty, Boari flap, renal autotransplantation, appendiceal interposition and ileal interposition grafting) and interventional techniques [2]. While various surgical and endoscopic options exist for treating ureteric strictures, selecting the right approach based on the stricture's characteristics is critical.

This manuscript presents a case of a long-segment ureteral stricture on the right side successfully treated using a minimally invasive endourological technique, preserving normal urinary flow, as the middle and distal thirds of the ureter remained intact. This is the first study to report a successful synergistic interventional approach for managing complex ureteral strictures.

## Case presentation

A 50-year-old diabetic woman with chronic obstructive pulmonary disease (COPD) presented with fever, loin pain, and vomiting. She had previously been treated for acute pyelonephritis, during which an attempt was made at bilateral ureterorenoscopy and double J (DJ) stenting. While the stent was successfully placed on the left side, the procedure on the right kidney could not be completed.

We performed axial imaging with computed tomography (CT) upon presentation to our center.

**Figure 1:**
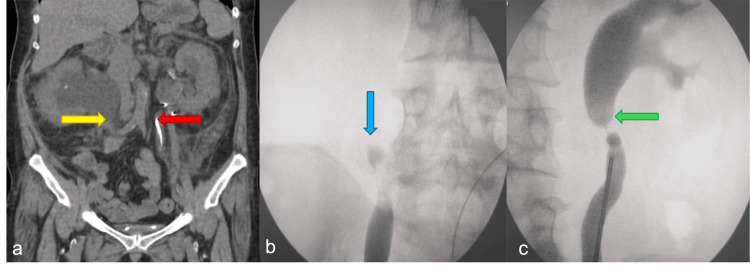
CT image (a) and fluoroscopic images of both kidneys (b, c) (a) Right-sided pelvi-ureteric junction obstruction/stricture(yellow arrow). (b) Blind ending ureter with no contrast passage across the narrow segment observed on the right side (blue arrow). (c) Narrowing at the L3 level of the ureter on the left side (green arrow).

Figure 1 illustrates the CT and retrograde ureterogram images of the right and left kidneys. Figure 1a demonstrates the coronal reconstruction CT image with a right-sided pelvi-ureteric junction (PUJ) obstruction/stricture (yellow arrow) and a downward-migrated DJ stent on the left side (red arrow). Attempts to place a stent on the right side were unsuccessful due to a blind-ending ureter, with no contrast passage across the narrow segment observed (Figure 1b, blue arrow). A right percutaneous nephrostomy (PCN) was performed to preserve the right renal function, and a 6 Fr ureteric catheter was placed at the site, as demonstrated in the retrograde pyelogram (RGP) image.

An RGP on the left side revealed a narrowing at the L3 level of the ureter, which was negotiable with a ureteroscope and Terumo guidewire (Terumo Corporation, Tokyo, Japan) (Figure 1c, green arrow). Consequently, the patient was successfully stented on the left side. Given her history of chronic kidney disease (CKD), she underwent multiple cycles of hemodialysis to regulate renal parameters.

**Figure 2:**
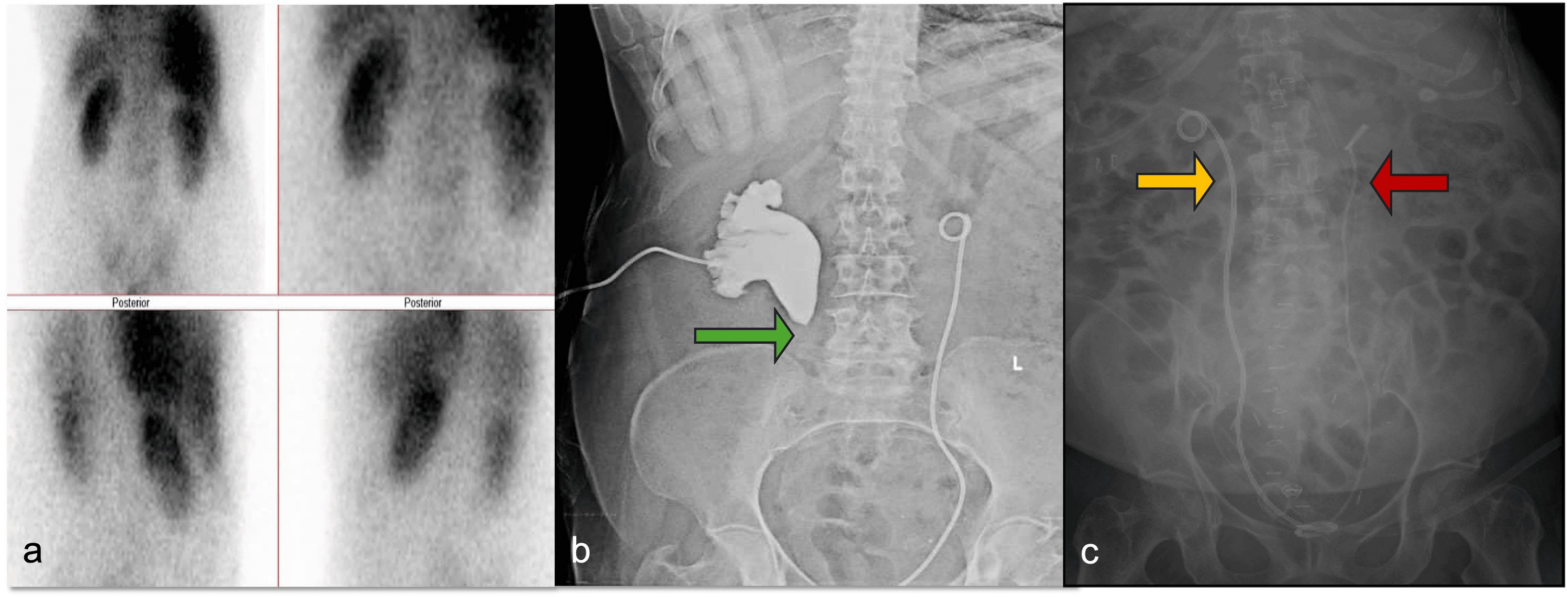
Nuclear imaging (a) and X-ray images of the kidney, ureter, and bladder (KUB) (b, c) (a) Bilateral renal scarring with reduced renal function. (b) Nephrostogram showing complete ureteral occlusion at the pelvi-ureteric junction level (green arrow). (c) Kidney, ureter, and bladder (KUB) X-ray showing 8 Fr stent on the right side (yellow arrow) and 6 Fr stent on the left side (red arrow).

To further assess renal function, a dimercaptosuccinic acid (DMSA) renal scan was performed following the right PCN placement. Figure 2a illustrates bilateral renal scarring with reduced cortical function (52% function in the left kidney, 48% in the right kidney). After three weeks, an antegrade nephrostogram demonstrated complete ureteral occlusion at the PUJ level (Figure 2b). Considering the patient's multiple comorbidities and Eastern Cooperative Oncology Group (ECOG)-2 performance status, a minimally invasive endourological approach was preferred over renal descensus and ureteropyeloplasty, as she was deemed unfit for a prolonged surgical procedure.

The patient was initially planned for laser ureterotomy through a rigid ureteroscope, but we were not able to get a guidewire across the proximal stricture segment; hence, we had to perform a nephroscopy through the already placed right PCN tract. Through the combined nephroscopy and ureteroscopy, we were able to perform Endoscopic Combined Intrarenal Surgery (ECIRS) with holmium laser. The distal stricture was managed with a medial ureterotomy, while the proximal end of the stricture was treated using the "cut-to-light" technique with a lateral laser ureterotomy. Following the procedure, an 8 Fr stent was placed, as the 7/14 endopyelotomy stent was not negotiable (Figure 2c).

Post-operatively, the patient recovered well; we were able to see improvements with regard to pain score and renal parameters. She was discharged on the fifth post-operative day. The patient remains under regular follow-up with plans for stent removal, followed by RGP and ureteroscopy to assess the outcomes of the laser endoureterotomy.

## Discussion

Following the advent of ureteroscopy, endo-urological interventions have become the primary cause of ureteral strictures, surpassing gynecology and surgery. Nearly 3% of patients undergoing ureteroscopy develop ureteric strictures over time [3,4]. A significant number of such strictures end up in nephrectomy. A retrospective review of 40 patients with post-ureteroscopic strictures revealed that one-fourth of such patients needed nephrectomy eventually [5]. The underlying causes include calculus passage, mechanical trauma, perforation, ischemia due to large-caliber ureteral instruments, and thermal injury [6,7]. Before the adoption of minimally invasive techniques, various surgical approaches were employed, including renal descensus, pyeloplasty, TUU, buccal mucosal graft ureteroplasty, bowel interposition grafts, the Boari flap, and renal autotransplantation [8]. These surgical procedures were associated with extended hospital stays, increased morbidity, and elevated treatment costs.

Popescu first described renal descensus surgery in 1964 [9]. This procedure was recommended for ureteral injuries or strictures measuring approximately 8 cm in length. However, it may not be a suitable option for patients with anatomically short renal vessels, which could necessitate renal autotransplantation. In cases of short proximal ureteric strictures, ureteroureterostomy is preferred, requiring multiple maneuvers to achieve an optimal ureteral anastomosis length.

TUU is another surgical option wherein a retroperitoneal tunnel is created, and the donor ureter is anastomosed to the recipient ureter in an end-to-side fashion [10]. However, this approach is limited to proximal ureteric strictures near the sacral margin. The extensive mobilization and dissection involved in this procedure increase the risk of recipient ureteral strictures.

For long-segment proximal or bilateral strictures, bowel interposition grafts provide adequate length. Both ureters can be anastomosed to a single bowel loop and connected to the bladder. However, this procedure is contraindicated in patients with hepatic dysfunction and azotemia.

The appendix can also serve as a bowel segment interposition graft for ureteral replacement due to its similar caliber to the ureter. However, its short length limits its application in terms of both size and anatomical side. Xiong et al., in their review paper on intestinal interposition options for complex ureteral strictures, stated that such interpositions (appendiceal onlay ureteroplasty) are greatly amenable for right-sided ureteric strictures [11]. They also concluded that the larger caliber and mucosal surface make the colon another viable option in patients with long-segment ureteral defects. Renal autotransplantation remains a viable option for complex cases.

Balloon dilatation as a treatment for ureteral strictures was first documented by Banner et al. in 1983. In the same year, Wickham introduced an endoscopic approach for treating ureteropelvic junction obstruction using a cold-knife urethrotome combined with a PCN tube [12].

Since then, several endoscopic techniques have been explored, including laser endoureterotomy, electrocautery, cold- or hot-knife incision, and stenting. Cold-knife ureterotomy, which avoids thermal damage to surrounding tissues, is restricted to the distal ureter due to the requirement of a large-caliber scope, which is often difficult to maneuver within the ureter [13].

Laser energy has been identified as a superior option over cold-knife ureterotomy due to its combined hemostatic and cutting capabilities [14]. Additionally, it is compatible with both rigid and flexible endoscopes of all sizes, ensuring a clear surgical field while providing simultaneous hemostasis and precise cutting.

Holmium:yttrium, aluminum, and garnet (Ho:YAG) laser has a wavelength of 2140 nm and pulse duration of 350 ms. It is a pulsed type of laser with a depth of penetration of 0.4 mm and also causes rapid coagulation of small and medium-sized vessels to a depth of about 2 mm. It is used for the prostate, laser lithotripsy, ablation of urothelial tumors, and upper and lower urinary tract strictures. The Thulium (Tm):YAG laser has a 2000 nm continuous wavelength. Since it has a short wavelength, the depth of penetration is only up to 0.25 mm. It can function in pulsed mode for precise cutting or in continuous-wave mode for enhanced hemostasis and coagulation. We went with a choice of Ho:YAG laser as it had better depth of penetration and depth of coagulation [15,16].

Table 1 presents an overview of the comparison between the traditional and endoscopic procedures.

**Table 1:** A side-by-side comparison of traditional and ECIRS approaches ECIRS, Endoscopic Combined Intrarenal Surgery; YAG, yttrium, aluminum, and garnet

Parameter	Traditional Surgical Approaches	Endoscopic Synergy (ECIRS-Guided "Cut-to-Light" Holmium Laser Endoureterotomy)
Indication	Long-segment ureteral strictures requiring extensive reconstruction	Long-segment ureteral strictures, particularly in high-risk or comorbid patients
Techniques Utilized	Renal descensus, pyeloplasty, transureteroureterostomy, buccal mucosal graft ureteroplasty, Boari flap, renal autotransplantation, appendiceal/ileal interposition	ECIRS with a Holmium:YAG laser employing the "cut-to-light" technique
Surgical Complexity	High, involving major reconstructive procedures with extensive tissue dissection	Minimally invasive, leveraging dual endoscopic access for precise intervention
Operative Morbidity	Elevated risk of complications, including bleeding, infection, and prolonged recovery	Reduced morbidity, minimal bleeding, and lower complication rates
Hospital Stay and Recovery	Extended hospitalization and rehabilitation period	Shorter hospital stay with faster postoperative recovery
Anatomical Considerations	Requires significant tissue mobilization and reconstruction	Preserves native ureteral structure with targeted intervention
Tissue Preservation	Potential for collateral damage due to extensive surgical manipulation	Minimal tissue trauma with precise laser incision and hemostasis
Renal Function Preservation	Variable, dependent on ischemia risk and surgical success	Enhanced preservation due to minimal invasiveness and controlled energy delivery
Applicability	Preferred for complex strictures with extensive ureteral involvement	Optimal for patients with focal or long-segment strictures unsuitable for major surgery
Postoperative Management	Routine follow-up for potential anastomotic strictures and complications	Requires post-procedure retrograde pyelogram (RGP), ureteroscopy, and stent removal follow-up
Technological Requirements	Conventional surgical instruments and open/laparoscopic techniques	Advanced endoscopic technology, Holmium:YAG laser, and fluoroscopic guidance
Success Rate	High in carefully selected cases but may necessitate revision surgery in complex scenarios	Favorable outcomes in selected patients, particularly those unfit for open reconstruction

Initial plan for regular ureterotomy laser in this patient 

Stricture length significantly impacts the success of endoureterotomy [17]. Shorter strictures generally demonstrate better outcomes with endoscopic techniques, whereas longer strictures may necessitate alternative surgical or reconstructive approaches, but in our case report, since the patient could not undergo a reconstructive procedure, we employed the use of the cut-to-light technique for endoureterotomy.

Our initial plan for a standard ureterotomy using a laser could not proceed because the guidewire could not be successfully advanced. Upon making a laser incision in the ureter, we encountered retroperitoneal fat, indicating a high risk of perforation. Continuing with the procedure at that point could have resulted in incomplete treatment and potential complications. Therefore, we had to modify our approach accordingly.

Key takeaways

ECIRS with a Holmium laser offers effective stricture management while reducing recovery time and surgical morbidity. This approach helps maintain renal function, particularly in patients with complex strictures and multiple comorbidities. Additionally, laser endoureterotomy provides a viable, less invasive option for patients who are not candidates for major surgical repair.

## Conclusions

ECIRS, combined with ureteroscopy, offers a practical and efficient minimally invasive approach for managing long-segment juxta-PUJ ureteral strictures. This cut-to-light technique offers a viable alternative to conventional major reconstructive surgeries by enabling precise tissue incision and effective hemostasis while minimizing collateral damage to surrounding tissues. Such dual-access strategies leverage the advantages of both techniques, leading to improved clinical outcomes, reduced complications, and effective management of complex ureteral strictures. Though it has been performed for a single case and cannot be a standard care yet, as it needs a good sample size to assess its efficacy, also the lack of long-term follow-up data is a drawback in the case.
